# Developing a community-led rare disease ELSI research agenda

**DOI:** 10.1186/s13023-023-02986-x

**Published:** 2024-01-22

**Authors:** Courtney Berrios, Macy McBeth, Andrea Bradley-Ewing, Nikolaus Schuetz, Annette Campbell, Zohreh Talebizadeh, Jeremy R. Garrett, Tamara Falicov, Francisco Martinez, Rare Voices Advisory Group, Jensen Adams, Jewel Akpan, Kelly L. Baesel-Freund, Urvi Biyala, Emily Cramer, Kimberly Freeman, Terrence Gallagher, Ada Jarrar, Linda Vaughn Jones, Tamorah Lewis, George Long, Nancy Petersen, Kerry K. Prout, Alejandro Rosas, Meghan Strenk, Melissa Wirtz, S. Margaret Wright, Miller Wood, West Wood, Emily A. Hurley

**Affiliations:** 1 Genomic Medicine Center, Children’s Mercy Kansas City, Kansas City, MO USA; 2 University of Missouri Kansas City, Kansas City, MO USA; 3 Health Services and Outcomes Research, Children’s Mercy Kansas City, Kansas City, MO USA; 4 RareKC, Overland Park, KS USA; 5 American College of Medical Genetics and Genomics, Bethesda, MD USA; 6 Bioethics Center, Children’s Mercy Kansas City, Kansas City, MO USA; 7 Office of Equity and Diversity, Children’s Mercy Kansas City, Kansas City, MO USA; 8 Department of Population Health, Kansas University Medical Center, Kansas City, KS USA

**Keywords:** Rare disease, Community engagement, Research agenda, ELSI

## Abstract

**Background:**

Research priorities are best defined through engagement with communities who will be impacted by the research and have lived experience of the topics to be studied. We aimed to establish a pediatric rare disease community stakeholder group and empower them in (1) eliciting perspectives from affected families in the wider region and (2) synthesizing collective ideas into a research agenda focused on shared ethical, legal, and social implications (ELSI) across rare disease.

**Methods:**

This two-year project utilized a community-centered approach to engage rare disease community members as equal partners in developing a research agenda for ELSI in rare disease. We established “Rare Voices” (RV), a 22-member stakeholder group of patients, parents, clinicians and researchers. Following capacity-building trainings, RV designed and conducted listening sessions with teen patients and parents of children with rare diseases to explore challenges, positive experiences, and ethical concerns. Listening session findings were synthesized and contextualized into research topics, which RV members further refined and prioritized. We used established measures to assess RV member engagement and satisfaction.

**Results:**

From 14 listening sessions with parents (n = 52) and teen patients (n = 13), RV identified eight core research topics as most important for future rare disease research: coordinating care, communication, accessing resources and care, impact on family unit, community and support in society, mental health and identity, ethical aspects of care, and uncertainty. RV members were highly engaged throughout the two-year project and reported high levels of satisfaction with the experience and research agenda.

**Conclusions:**

Through capacity-building and authentic engagement, this project resulted in a community-led rare disease research agenda to guide future rare disease ELSI research that aligns with patients’ and families’ priorities. An environment of equal partnership and respect created a space for mutual learning where community members were empowered to shape the research agenda based on their collective experiences. The agenda recognizes the shared psychosocial and healthcare experiences of rare disease and offers practical areas of research to address patient and family needs.

**Supplementary Information:**

The online version contains supplementary material available at 10.1186/s13023-023-02986-x.

## Background

Community engagement throughout all stages of the research process is recognized to enhance study relevance and quality [1–3]. Reflecting this, community engagement in research is increasingly encouraged and even required by funders [4]. In rare disease research, communities of patients, families, and advocates are assuming increasingly influential roles [5]. Members of these communities contribute as partners in obtaining funding, designing studies, recruiting participants, collecting data, and disseminating findings [6, 7]. Within disease-specific advocacy organizations, community members may even take the lead in directing research studies. Yet despite their involvement in these activities, patients and families are less often engaged in shaping a research agenda for a field of study.

While there is no standard methodology, a community-engaged research agenda-setting process commits to giving community stakeholders an equal role in knowledge creation from the earliest stages, countering the “epistemic injustice” that has historically reserved this privilege to researchers [8]. One approach, *responsive research,* seeks first to empower the least empowered stakeholders by providing them a platform to voice their priorities [9]. It then aims to integrate their priorities within a larger field of study by fostering dialogue with other types of stakeholders. At the heart of this process lies a focus on experiential knowledge, meaningful and sustained interaction between all stakeholders, mutual learning, openness, and respect [9]. Ideally, through sustained engagement, community stakeholders become empowered with a richer understanding of their collective priorities and a greater capacity to articulate them to stakeholders, including researchers and funders.

In the context of rare diseases, patient and caregiver communities have actively participated in research agenda-setting processes for specific diseases and groups of diseases [10–12]. The resulting research agendas have included some cross-cutting topics related to the patient experience, such as discrimination, relationships, community support, and psychosocial support [11, 12]. However, the scientific literature has yet to present a community-driven research agenda that reflects shared priorities of stakeholder communities across rare disease as a whole.

Cross-cutting topics in rare disease research agendas commonly address ethical, legal, and social implications (ELSI), a growing, multidisciplinary field. The ELSI Research Program established by the National Human Genome Research Institute has increased focus on ELSI in the field of genomics, which touches patients and families across the spectrum of rare and/or undiagnosed conditions [13]. Yet a growing recognition of the need to consider the patient experience as a whole has expanded the conceptualization of ELSI to include any challenge of living with rare disease that has ethical and/or psychosocial implications for patients and their families [14]. ELSI are often shared across rare diseases, irrespective of whether an underlying or genetic diagnosis is obtained [15–17]. Furthermore, the rarity of a disease can both exacerbate and bring a need for nuanced consideration to these issues [18]. For ELSI, leveraging the shared experiences across rare diseases can produce a more complete understanding of cross-cutting patient experience issues, overcome challenges of small sample sizes common to research on individual rare diseases, contribute to the development of shared outcome measures, and advance overall patient-centered care [19–21]. This holistic view of rare disease for research agenda setting may be particularly relevant for patients with ultra-rare and suspected, but undiagnosed, rare conditions, who are often overlooked when ELSI research focuses on specific diagnoses.

Research responsive to the shared priorities of patients and families with rare diseases is critical to advancing ELSI and patient-centered care. This paper describes a long-term patient and family engagement effort leading to a community-developed research agenda reflecting ELSI priorities across rare disease in the central region of the United States.

## Methods

### Overview

**Fig. 1:**
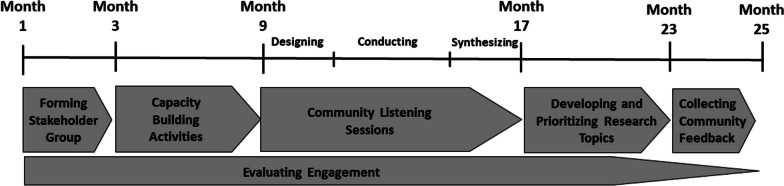
Overview of project design and timeline

In this 24-month project, we aimed to (1) establish a group of rare disease stakeholders (patients, families, clinicians and researchers) and (2) support the group in developing a research agenda reflective of the ELSI needs of patients and families living with rare diseases in the central region of the United States (defined for this project as the states of Missouri, Kansas, Nebraska, Iowa, Illinois, Kentucky, Tennessee, Arkansas, or Oklahoma). Figure 1 provides an overview of the project design. The stakeholder group was formed and guided by a ten-member Project Core (PC) consisting of a lead patient family advisor and researchers with experience in rare disease, genomics, health outcomes, community engagement, and ethics. The stakeholder group (called *Rare Voices* [RV]) participated in capacity-building activities, conducted listening sessions to elicit perspectives from their wider community, then synthesized their perspectives and the listening session findings into a prioritized research agenda. The project was reviewed by the Institutional Review Board at Children’s Mercy and determined not to be human subjects research.

### Rare disease stakeholder group: Rare Voices

Parent and teen stakeholders representing patients and family members were recruited through a local rare disease advocacy group, patient and family advisory councils at our institution (a pediatric academic medical center), and clinics at our institution that frequently care for patients with rare conditions. A project information sheet directed interested individuals to a survey in REDCap [22, 23] collecting contact information, demographics, information about their/their child’s rare diagnosis, and availability for stakeholder group meetings. The PC then selected members who represented diversity in rare disease diagnosis or lack of a diagnosis, age, age of affected child (parents only), gender, race or ethnicity, and residence in rural, suburban, or urban areas. Clinician and researcher members were identified through professional connections of the PC and selected to represent diverse specialties, race, and ethnicity.

Selected members included 12 parents of children with rare conditions, 4 teens with experiences related to rare conditions, and 6 clinicians and/or researchers with specialties including genetic counseling, neonatology, primary care for children with complex medical needs, child psychology, and quantitative psychology. This categorization reflects the primary role for which each stakeholder was selected. Some stakeholders identified with multiple roles (e.g. parent and nurse). Parents and teens represented 11 different rare disease diagnoses and 2 parents had children with suspected, but undiagnosed, rare conditions. All conditions represented include physical manifestations affecting various body systems, a substantial minority also include cognitive impacts. RV members were 77% female and 82% identified as White, 14% as Black or African American, 9% as Hispanic or Latinx, and 5% as Asian. Half had a graduate degree and another 23% had a bachelor’s degree (see Table 1 for full demographics).

**Table 1:** Demographics for Rare Voices members, listening session participations, and individuals who completed the survey for rare disease community feedback on the research agenda

Participant characteristic	Rare voices stakeholders (n = 22) n (%)	Listening session participants (n = 65) n (%)	Community feedback survey participants (n = 37) n (%)
Age (years)^a^			
< 20 years	4 (18%)	13 (20%)	4 (11%)
20–29	1 (5%)	9 (13%)	4 (11%)
30–39	8 (36%)	18 (28%)	11 (30%)
40–49	6 (27%)	17 (26%)	12 (32%)
50–59	2 (9%)	5 (8%)	4 (11%)
60 years or older	1 (5%)	2 (3%)	2 (5%)
Prefer not to answer	0 (0%)	1 (2%)	0 (0%)
Age of Participants’ Affected Child/Children^b,c^			
0–5	3 (25%)	20 (38%)	N/A
6–10	4 (33%)	9 (17%)	N/A
11–20	3 (25%)	19 (36%)	N/A
21 and above	2 (17%)	5 (9%)	N/A
Deceased	2 (17%)	2 (4%)	N/A
Unknown	0 (0%)	2 (4%)	N/A
Gender			
Female	17 (77%)	55 (85%)	33 (89%)
Male	5 (23%)	10 (15%)	4 (11%)
Education			
Less than high school	3 (13.5%)	7 (10%)	N/A
High school degree	3 (13.5%)	16 (25%)	N/A
Associates degree or some college	0 (0%)	2 (3%)	N/A
Other post-high school training	0 (0%)	5 (8%)	N/A
Bachelor’s degree	5 (23%)	15 (23%)	N/A
Graduate degree	11 (50%)	20 (31%)	N/A
Race^d^			
American Indian/Alaskan Native	0 (0%)	3 (4%)	1 (3%)
Asian	1 (5%)	5 (7%)	1 (3%)
Black/African American	3 (14%)	6 (9%)	1 (3%)
Native Hawaiian/Other Pacific Islander	0 (0%)	0 (0%)	0 (0%)
White	18 (82%)	54 (83%)	32 (91%)
Other	0 (0%)	2 (3%)	0 (0%)
Unknown/prefer not to answer	(0%)	1 (2%)	3 (8%)
Ethnicity			
Hispanic	2 (9%)	5 (8%)	4 (11%)
Not Hispanic	20 (91%)	58 (89%)	32 (86%)
Unknown/Prefer not to answer	0 (0%)	2 (3%)	1 (3%)
Area Living			
Rural	7 (32%)	23 (36%)	N/A
Urban	3 (14%)	32 (49%)	N/A
Suburban	12 (54%)	10 (15%)	N/A
Average Annual Income^e^			
$0—$25,000	N/A	1 (2%)	N/A
$25,001—$50,000	N/A	7 (14%)	N/A
$50,001—$75,000	N/A	9 (17%)	N/A
$75,001—$100,000	N/A	8 (15%)	N/A
Over $100,000	N/A	17 (32%)	N/A
Unknown/Prefer not to answer	N/A	10 (20%)	N/A
Support group participation^f,g^			
Yes	10 (63%)	35 (54%)	N/A
No	5 (31%)	29 (44%)	N/A
Unknown	1 (6%)	1 (2%)	N/A

a) Rare Voices stakeholder age at time of project start, b) data not applicable for clinicians/researchers and teens in both stakeholder and listening session participants, c) percentages do not add up to 100% because participants may have more than one affected child, d) percentages may not add up to 100% because some individuals identified as more than one race, e) data (US dollars) was not collected for Rare Voices members, feedback survey participants, or teen participants in listening sessions (the median household income in the US in 2021 was $70,784) [33], f) data not applicable for clinicians/researchers in Rare Voices, g) data not collected for feedback survey participants

RV met monthly, with additional ad hoc meetings of small teams to accomplish tasks as needed. Most meetings were held virtually on a video-conferencing platform due to the COVID-19 pandemic. After COVID-19 restrictions lifted, three hybrid virtual/in-person meetings were held to facilitate more informal interactions and relationship-building while maintaining a virtual option. All meetings were recorded so members not able to attend could watch and provide input. RV members were compensated for participation in all project activities.

### Capacity building activities

The first six months were devoted to capacity-building activities, including planned topics and training needs identified by RV members. RV members received a project onboarding manual (modeled after established tools for community engagement [24]) and completed institutional training on conduct, confidentiality, and conflicts of interest. RV members then completed modules on community engagement and developing research questions from the Patient-Centered Outcomes Research Institute’s (PCORI) Research Fundamentals [25]. Further preparation for conducting community listening sessions included an introduction to qualitative methods and interactive training in developing research questions.

Based on RV members expressing struggles with revisiting difficult experiences through the project, a trauma workgroup of RV members was formed. The workgroup completed a trauma-informed care training, led the training for the larger RV group, and developed guidance for incorporating trauma-informed practices into RV and listening session processes. Also, at the request of parent members, a virtual meeting room for a parent-only meeting was made available after each monthly RV meeting to facilitate peer support and connection.

### Community listening sessions

#### Conducting listening sessions

RV members designed and conducted a series of listening sessions collecting views of challenges and research priorities from broad representation of the regional rare disease community. RV members were engaged in planning and conducting listening sessions via participation in three teams: the Recruitment Team, Guide Development Team, and Moderating Team.

The 5-member Recruitment Team developed recruitment materials (in English and Spanish) and strategy. Eligible listening session participants were teen patients or caregivers of children who lived in the central region of the United States and self-identified with terms relevant to rare disease including “rare,” “genetic,” and/or “complex health concerns” (see Additional file 1 for word cloud used in recruitment materials). Recruitment materials were distributed in-person and virtually through local rare disease advocacy groups, clinics that commonly serve patients with rare diseases, other community clinics, institutional patient and family advisory groups, and posts on our institution’s social medial accounts. Supplemental efforts to improve recruitment of teens and racially and ethnically diverse participants utilized a list of patients and families from a genomic research repository primarily focused on rare diseases who had consented to be contacted for related projects.

The 5-member Guide Development Team drafted the discussion guide according to focus group methodology [26, 27]. Questions explored challenges, positive experiences, and ethical concerns (see Additional file 2 for discussion guide). Members of the Moderating Team (who completed supplemental training and practice sessions) conducted listening sessions. PC members with qualitative research expertise served as session note-takers, recording ideas and emergent themes as well as feedback on moderating technique.

Listening sessions were virtual and lasted approximately 90 min. Separate sessions were held for teens and parents/caregivers to facilitate comfort in sharing with peers. Participants completed a survey with demographics, their/their child’s rare diagnosis, age at symptom onset and diagnosis, and involvement in a support group before the session. PC members consented and scheduled all participants.

#### Synthesis of listening session data

A Synthesis Team of 4 RV members used a process informed by thematic analysis to identify common themes across the listening session data. The team reviewed notes and recordings from the first half of listening sessions and identified common themes that represented the emerging needs among common, shared experiences. These were then framed as research topics that could address each need. Each Synthesis Team member wrote a description of each topic of emerging needs they identified and listed subtopics. The PC lead identified shared topics across the descriptions and presented a master document of shared topics and subtopics to the Synthesis Team for revision. The team then reviewed the notes from the second half of listening sessions and suggested additions and revisions to the topics. These were again collated by the PC lead and integrated into a final list of eight topics and sub-topics approved by the full Synthesis Team.

### Producing a research agenda

#### Developing research questions

The PC completed a review of the pediatric rare disease literature related to each topic and sub-topic identified by the Synthesis Team. When literature specific to pediatric rare disease was inadequate, applicable literature from adult rare disease or medically complex children was utilized. The PC lead drafted a summary then considered knowledge gaps from the literature alongside descriptions of topics and sub-topics developed by the Synthesis Team to create a list of proposed research questions addressing the overall topic and each sub-topic.

Topic Teams of 2–3 RV members were formed to review each topic summary and proposed research questions. Team members then revised and added research questions based on their reading of the literature summary and personal viewpoints. Proposed research questions were presented to the full RV group for consideration and further revision.

#### Prioritizing research topics

RV members completed a survey to rank all eight topics by importance (“for children with rare diseases and their families”) and feasibility (“how difficult or easy it might be to have an impact”). For each topic, an optional, open-ended question asked, “In what ways are the other topics identified in this project related to [the topic]?” Respondents were asked to self-identify as a patient/teen, parent, rare disease advocate/community member, clinician, or researcher, where multiple options could be selected. Mean scores for importance, feasibility, and combined importance/feasibility were calculated for each topic. RV members voted to approve the final, prioritized agenda as reflective of the overall work of the group.

### Evaluating processes and outcomes

#### Collecting community feedback

A survey was used to seek feedback on the research agenda from the regional community of families affected by rare diseases. Respondents indicated on a 5-point Likert scale (strongly agree to strongly disagree) the extent that they agreed that research topics “represented the needs of rare disease patients and their families” and the extent they agreed with RV’s importance and feasibility rankings. Open-ended questions asked respondents to indicate what, if anything, they thought was missing from the research topics and to comment on the rankings. All listening session attendees were invited to complete the survey by direct phone and/or email contact. A local advocacy group for rare disease (spanning across diagnoses) also shared the survey via their email list and social media accounts.

#### Evaluating engagement

To assess engagement of RV members, we tracked attendance at monthly meetings, participation in small teams (recruitment, guide development, moderating, synthesis, topic review) and collected structured and unstructured feedback. Structured feedback was collected via surveys developed by PC and RV representatives and distributed at three timepoints: T1) after completion of capacity-building activities, T2) after completion of the listening sessions, and T3) at the end of the project. Each survey included the condensed version of the Research Engagement Survey Tool (REST) [28] and assessments of satisfaction with RV overall and with time commitment (5-point Likert scale, very satisfied to very dissatisfied). RV members also rated the success of RV in working together (4-point Likert scale, very good to poor), achieving goals (5-point Likert scale, strongly agree to disagree), and how often stakeholders felt their opinions were listened to and considered (5-point Likert scale, always to never). Open-ended items asked for aspects that were going well and that could be improved. The T2 evaluation included questions only for recruitment, guide development, moderating, and synthesis team members that asked if team participation increased knowledge or personal growth (5-point Likert scale, strongly agree to strongly disagree). The T3 evaluation assessed how well the research agenda represented the work of the group and each member’s own values and perspectives (4-point Likert scale, very good to poor) and an open-ended question about what was most impactful about their work with RV. Unstructured feedback was received during monthly meetings and via informal verbal or email discussions.

## Results

### Research agenda

#### Listening session findings

A total of 14 listening sessions (11 with parents/caregivers, 3 with teen patients) were completed, with a total of 65 participants (52 parents, 13 teens) from the rare disease community (see Table 1 for demographics). Overall, 75% of participants identified as White and not Hispanic or Latinx and 85% were female. The large majority (92%) resided within Kansas and Missouri, which our institution primarily serves. Participants represented experience with both diagnosed and suspected, but undiagnosed, rare conditions with diverse physical and cognitive symptoms.

The synthesis process identified eight research topics that would address the challenges and priorities of listening session participants: accessing resources and care, coordinating care, communication, community and support in society, ethical aspects of care, impact on the family unit, mental health and identify, and uncertainty. For each topic, three to seven sub-topics were identified.

#### Finalized research agenda

Twenty-one RV members completed the ranking survey (12 patient/family members; 9 who identified only as advocate/community members, clinicians and/or researchers). Table 2 presents the research topics and associated questions, as well as mean prioritization rankings (composite and stratified by respondent type). The prioritized research agenda was unanimously approved by anonymous vote at an RV meeting.

**Table 2:** Research topics identified by listening sessions with proposed research questions and the mean prioritization ranking score given by RV (n = 21) for importance, feasibility, and combined importance and feasibility

Research Topic	Proposed Research Questions	Mean Importance Ranking Score^a^ Overall^b^		Mean Feasibility Ranking Score^a^ Overall^b^		Combined Mean Importance and Feasibility Ranking Score^a^ Overall^b^	
		T/P^c^	A/C/R^d^	T/P^c^	A/C/R^d^	T/P^c^	A/C/R^d^
Coordinating Care	1. What outcomes of care coordination are most important to patients and families? 2. Who is responsible for care coordination? a. with internal providers? b. with external providers? 3. How do patient and disease characteristics impact needs and outcomes of care coordination? 4. What skill sets and values are required to be a care coordinator? a. perspectives of providers and parents 5. What care coordination activities are needed in different settings and with varying patient needs? a. age, medical needs, etc 6. What care coordination models and strategies most improve outcomes and relieve burdens for patients and families? 7. How can options and awareness of ways to sustainably pay for care coordination be raised?	2.19		3.43		2.81	
		2.58	1.67	3.08	3.89	2.83	2.78
Communication	1. What interventions/systems can remove barriers and promote communication between healthcare providers and community resources such as service agencies and schools? 2. What resources or training can help healthcare providers and schools coordinate care and communication? 3. How can existing technologies be used more consistently and applied in new ways to improve effective communication? a. among healthcare providers and between healthcare providers and other community/service providers including schools 4. How do we change healthcare culture to help facilitate optimal communication among care teams, patients, and their families?	4.05		3.52		3.79	
		4.58	3.33	4.67	2.00	4.63	2.67
Accessing Resources and Care	1. How can information about resources and services be more accessible and easily shared? a. resources and services include insurance, specialty providers, community services, financial resources, transportation services 2. What technologies can improve access to resources and care for families? 3. What technologies can improve access and delivery of care among providers between healthcare systems? 4. How can care and resources be provided in ways that minimize time, travel, and costs for patients and families?	3.05		4.90		3.98	
		3.00	3.11	4.92	4.89	3.96	4.00
Impact on Family Unit	1. What helps families come together in response to a rare disease diagnosis and through evolving developmental stages? 2. How is resilience (as related to experienced trauma) developed and supported in families impacted by rare disease? 3. What strategies effectively support siblings of children with rare conditions?	4.57		4.71		4.65	
		4.33	4.89	5.08	4.22	4.71	4.56
Community and Support in Society	1. What needs to be present for children with rare diseases to have full and engaged experiences in their developmental/educational processes? a. in various settings including school, daycare, community, etc 2. What is needed to facilitate or prepare individuals/organizations to provide appropriate accommodations for children with rare diseases? a. role of unconscious bias in receiving and the effectiveness of accommodations 3. What peer support activities or interventions improve health or psychosocial outcomes? a. role of information literacy in support group outcomes 4. How can we lower societal stigma associated with rare disease and increase acceptance of differences? a. tools for caregivers, advocates, places of work 5. How do we build empathy and advocacy in those not experiencing rare disease?	5.67		3.71		4.69	
		5.92	5.33	3.75	3.67	4.83	4.50
Mental Health and Identity	1. How can mental health interventions be tailored to patients and families impacted by rare disease? 2. How can mental health be integrated into healthcare delivery to support well-being for patients and families with rare disease? a. training healthcare providers outside mental health specialties to address patient and family mental health needs b. creating a warm and welcoming environment to reduce stress and increase openness to mental health discussions 3. How can children and adolescents share information about their condition in ways that are comfortable and retain their identity outside the condition?	4.67		5.00		4.84	
		4.67	4.67	4.58	5.56	4.63	5.11
Ethical Aspects of Care	1. How should quality of life be evaluated? a. inclusion, weighting, and balancing of patient, parent, and clinician views b. inclusion, weighting, and balancing aspects of quality of life i. biomedical, psychosocial, etc 2. How do clinicians balance patient and parent empowerment against their perceived best interests for the child? a. removing barriers to parent empowerment and advocacy b. managing expectations around influence in decision making processes 3. What disparities exist in rare diseases care based on socioeconomic and cultural factors? a. family resources, education, language, religious/cultural views, etc	5.62		4.43		5.03	
		4.83	6.67	3.75	5.33	4.29	6.00
Uncertainty	1. In what ways do children and adolescents with rare diseases experience uncertainty? 2. How can healthcare providers effectively communicate uncertainty to patients and families? 3. What coping strategies are effective for patients and families to manage uncertainty and improve emotional well-being? 4. How can healthcare providers reduce uncertainty or help patients and families manage uncertainty?	6.19		6.29		6.24	
		6.08	6.33	6.17	6.44	6.13	6.39

a) lower ranking scores reflect greater importance and feasibility, b) for each ranking the top bolded score is the overall ranking by all RV members, c) the lower left is the ranking score by RV members identifying as teens/patients/parents (T/P, n = 12), d) the lower right ranking score is by RV members identifying only as advocates/community members, clinicians, or researchers (A/C/R, n = 9)

The topics of coordinating care and uncertainty were ranked first and last, respectively, for both importance and feasibility (Table 2). Other topics varied in their rankings by importance and feasibility. Rankings by respondent type differed slightly, with the two groups differing by > 1 full ranking point for communication and ethical aspects of care.

According to open-ended survey responses, respondents perceived numerous interconnections among topics. Across the 21 surveys, each topic was linked to every other topic at least once. Multiple members stated that all topics were linked, noting that improvements in one topic could yield improvements in others. Coordinating care and communication were frequently connected to one another and were most frequently identified as the topics connected to all others.

#### Community feedback

A total of 35 community members completed the feedback survey, 31 of whom were listening session participants. Demographics for survey respondents are provided in Table 1 and survey results are provided in Fig. 1. All respondents either strongly agreed (65%) or agreed (35%) that the research topics represented the needs of rare disease patients and their families. A majority also agreed with the rankings by mean importance (73% strongly agree or agree) and feasibility (78% strongly agree or agree), though opinions on the rankings were more variable. Among comments regarding ranking, the most common theme was that mental health and identity should be ranked higher in terms of importance.

### Evaluating engagement metrics

**Fig. 2:**
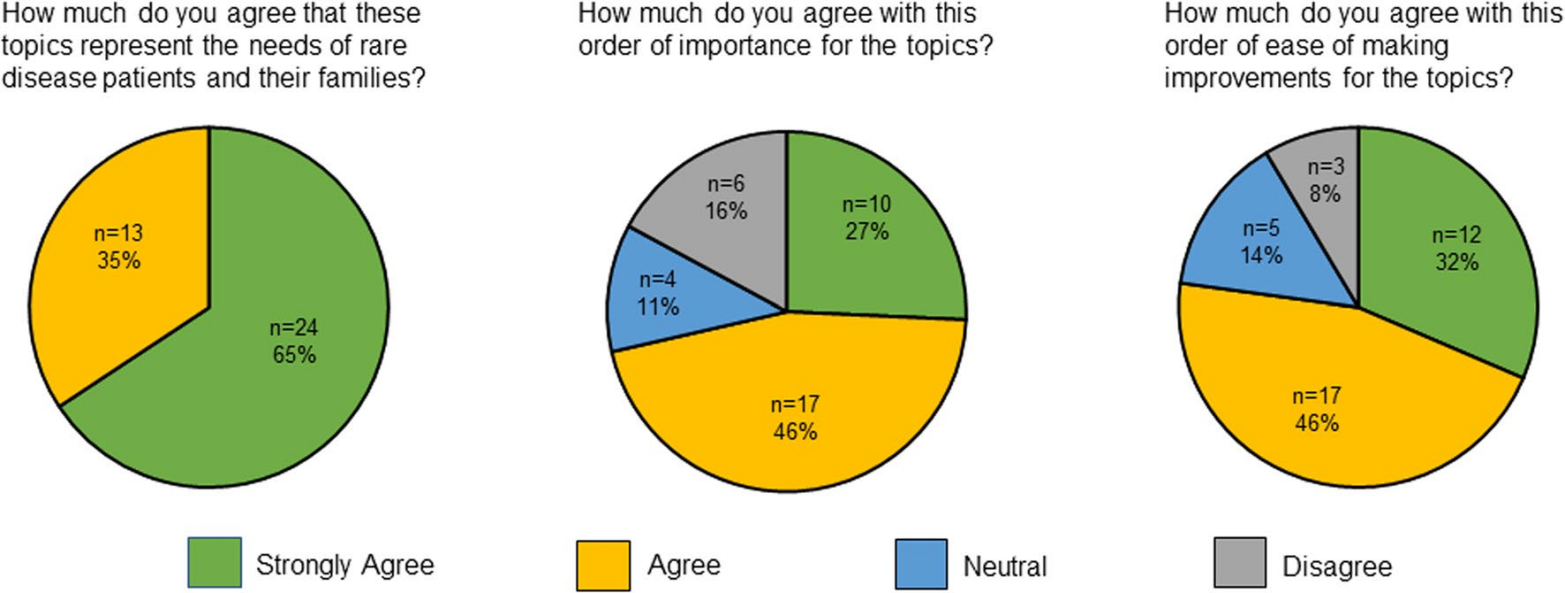
Community feedback survey responses (n = 37) indicating agreement with research agenda developed by Rare Voices

RV members remained engaged throughout the project. On average, 15.6/22 (71%) of RV members either attended or watched a recording and sent feedback for each monthly meeting. A total of 15/22 (68%) of members were involved in the small teams for recruitment, guide development, moderating, or synthesis and 11/22 (50%) joined one or more topic team to draft research questions (Fig. 2).

One parent left RV with approximately 6 months left in the project due to conflicting commitments. While no teen members resigned from RV, engagement was challenging. Teens reported that meeting times often conflicted with activities such as sports and other extra-curricular activities. The offering of text communications and all-teen small group discussions during RV meetings, which were suggested by teens, did not impact teen engagement.

**Fig. 3:**
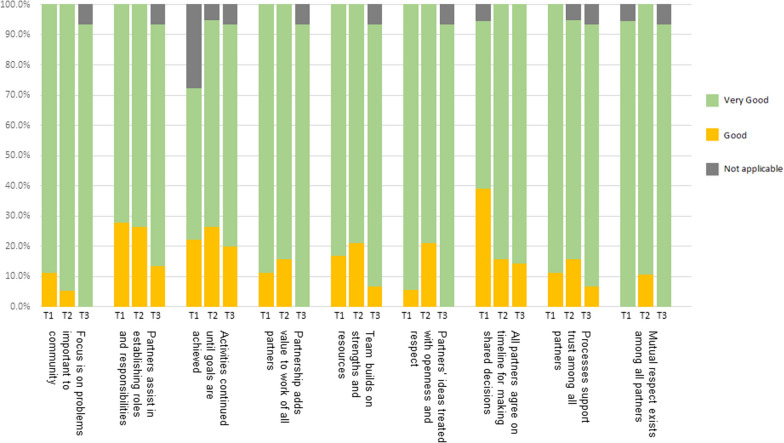
Results of RV engagement evaluation responses to the 9 items on the condensed REST using a 4-point Likert scale (very good to poor) and an option for not applicable for each item. T1 = after RV training was complete (n = 18), T2 = after listening sessions were completed (n = 19), T3 = at project completion (n = 15)

Results for the condensed 9-item REST survey across the three timepoints are presented in Fig. 3. Most RV members responded that engagement was “very good” or “good” for all items across all timepoints. Free text responses prompted improvements to project processes, including more time for small group discussions. At T2, 80% of RV members involved in teams that planned and conducted listening sessions agreed or strongly agreed that they had increased their knowledge and experienced personal growth due to their involvement. At completion of the project (T3), all respondents felt that the group was effective in achieving its goals (33% agree, 67% strongly agree), the group worked well together (13% good, 87% very good), and their opinion was listened to and considered (80% always, 20% often). Respondents were also satisfied with the time commitment (47% satisfied, 53% very satisfied) and their overall participation in RV (20% satisfied, 80% very satisfied). All RV members completing the T3 survey also responded that the priorities in the research agenda represented the work of the group (92% very good, 8% good) and their own values and perspectives (77% very good, 23% good). Free responses to what was most impactful for members included having a safe and respectful space to share views and feel heard, a feeling of community, and learning from other perspectives.

## Discussion

This community engagement project aimed to collaboratively develop an ELSI research agenda for pediatric rare disease that reflects priorities of patient, family, clinician and researcher stakeholders. The project employed a *responsive research* [9] approach, where stakeholders were empowered through capacity-building and involved as equal partners from the onset. While disease-specific research agendas have been produced using community-engaged methods [10–12, 29], to the best of our knowledge, this is the first community-led research agenda that focuses on ELSI topics and the patient and family experience across all rare diseases. Our process successfully engaged members throughout two years and produced a research agenda that acknowledges the psychosocial impact of rare disease and identifies practical areas of improvement for healthcare and quality of life of patients and families.

Our engagement efforts were regionally focused, which is both a strength and limitation. The research agenda is not intended to be generalizable beyond the U.S. central region, a largely rural area with significant geographical distances between pediatric and rare disease specialty care centers. However, the regional perspective gives increased local relevance to the research agenda and other results of our engagement efforts. With local dissemination and advocacy, the agenda can inform research and drive improvements within health systems that are most relevant to the specific contexts in which patients and families have encountered the challenges they identified.

While the phenotypic diversity in rare diseases can yield distinct needs for clinical research, this project successfully created an ELSI research agenda that reflects shared priorities across diagnoses or conditions. The resulting priorities focus largely on the healthcare and social challenges of living with rare disease and do not explicitly include human subjects research and technology-focused issues that are common in ELSI research (e.g. data collection, data sharing, and consent) [14]. As the PC did not want to direct the group to specific ELSI topics, the resulting priorities underscore the overall saliency of psychosocial and care experience to stakeholders.

Reflecting responsive methodology [9], the project emphasized empowerment of the least empowered stakeholders, as parents and patients represented the majority of RV. Meanwhile, the mixing of patients, parents, clinicians, and researchers into shared small teams and group discussions facilitated mutual learning and mobilization of community members’ strengths, key principles of community engagement [9, 30]. RV members consistently stated that small group work was key to having all members provide input and PC and RV members frequently mentioned seeing new complexities in topics as they listened to others’ perspectives. While stakeholder subgroups slightly differed in their prioritization rankings (see Table 2), the combining of rankings of these different groups is a strength of this project, as they incorporate and report views of multiple stakeholder groups while remaining weighted to the patient/family perspective due to their majority representation. Yet considering the low precision of the ranking (due to RV members' expressions of difficulty in the task as well as the interconnectedness of the topics) we place emphasis on the research agenda as a whole over the prioritization. This is further supported by our community feedback on the research agenda which showed less agreement on the prioritization of topics than on the overall, un-ranked topic list.

Finally, sustained stakeholder engagement throughout the project was facilitated by openness and mutual respect, which RV members rated as high in evaluations throughout the project. A strength of this project was regular and standardized engagement evaluations, which other engagement projects have often lacked [3, 6]. While REST items may have shown ceiling effects based on the condensed scale [28] and motivated RV members, both structured and unstructured feedback allowed the PC to recognize processes supporting engagement and to make improvements.

### Limitations

Limitations of the project include that a 22-member stakeholder group cannot represent all perspectives in rare disease, though this was addressed through collecting broader views in listening sessions and community feedback. Additionally, a majority of RV members and participants in listening sessions and community feedback identified as White and highly educated. Though discussions during RV meetings and listening sessions suggested shared experiences across socio-economic groups, we recognize that limited representation could have led us to miss the experiences of some communities, which may have produced a final research agenda that emphasized, interpreted, and/or defined topics differently. In particular, we did not successfully recruit any listening session participants who were not fluent in English, although 15.4% of our local metropolitan population reports Hispanic or Latino origin [31] and 2.6% of Missourians are Spanish-speaking (0.9% speak Spanish with limited English) [32]. Potential cultural differences in language used and lack of pre-existing relationships with Hispanic and Latinx organizations may have limited the ability of the RV group to reach Spanish-speaking participants [30]. We also struggled to consistently engage teen RV members and had fewer listening sessions with teens. While the Synthesis Team took care to pull themes specifically emphasized by teens into the research topics (e.g., mental health, identity, and community interactions), teens were also under-represented in the prioritization of topics by RV. Therefore, the research topics and priorities may have differed with greater representation of teens and there may be value in repeating this process with only teen stakeholders.

## Conclusions

Our work demonstrates the power of responsive community engagement and mutual learning in creating a research agenda that represents the patient and family experience across rare diseases. The agenda in turn can guide patient-centered research that will address the ELSI and care challenges that are most important to patients and their families in our region. Despite the local focus of our engagement, we expect many of the overarching topics will be important to rare disease patients and families beyond our region based on their overlap with other literature [15, 16]. However, as prioritizations and specific proposed research questions may well differ by region and population, replication of these engagement methods in other localities and groups is needed. Additionally, application of these methods with both pediatric and adult patient stakeholders will clarify ways that priorities may differ between patients and caregivers and based on patient age. Through engaging communities in research-agenda setting, the rare disease field will both improve the relevance and quality of research and empower community stakeholders to serve as partners throughout the research process.

### Supplementary Information

**Additional file 1**: Example listening session recruitment material posted on social media that demonstrates a word cloud of terms related to rare disease developed by the Recruitment Team

**Additional file 2**: Discussion guide utilized for listening sessions

## Data Availability

The datasets used during the project are available from the corresponding author on reasonable request.

## Abbreviations

ELSI: Ethical, legal, and social implications
RV: Rare voices
PC: Project core
