# Supplementary material for: Developing a community-led rare disease ELSI research agenda
Source: Orphanet J Rare Dis. 2024 Jan 22;19:23. doi: 10.1186/s13023-023-02986-x (PMC10801933; doi:10.1186/s13023-023-02986-x)
Supplement: Supplementary file 2 — Additional file 2: Discussion guide utilized for listening sessions [file 13023_2023_2986_MOESM2_ESM.pdf]

Additional file 2. Discussion guide utilized for listening sessions.

Instructions for using guide:

Text in *italics* is for moderator background. When wording should differ between parent/caregiver and teen sessions, both phrasing options are provided in brackets [ ]. While this is a semi-structured guide, areas in the introduction where moderators are specifically invited to use their own wording or share about themselves are in parentheses ( ).

Introduction

Greet people as they enter the virtual room

Hi everyone. Welcome to this listening session. We invited you each today because we want to you to share (your story/experience about your journey) so that the research being done on rare diseases is centered on what is important to [us as parents / patients with rare diseases]. So today, we're all gathered for the purposes of sharing our opinions on what topics related to rare conditions in children are most important so that research done in Children's Mercy and beyond can be centered on what is most important to us.

My name is __________ and I going to be facilitating this discussion tonight. I am a (parent of a child with a rare disease, just like you, who has been down this road… keep it simple**).** I am joined by____________ who is (hospital staff: making the point that they are taking the notes, listening, be the extra set of ears, represents the importance of these listening sessions to institution).

While I have some questions I want to ask, my job is really to listen. There are no right or wrong answers, we are really just interested in learning about your personal opinion. We want to create a space where everyone has the opportunity to share. And remember, you never have to answer a question you feel uncomfortable, and you are free to leave the discussion any time you choose.

We are going to audio record today's conversation so we can get down everything everyone says, and then produce a transcription of the recording. We will keep the recording confidential, delete it when we have a good transcription, and make sure your name is not attached to the recording or transcription. We hope too that you respect the privacy of everyone in this group and keep what is discussed within this session.

I'm going to let __________ tell you a little about resources you can access if you may need them. *Staff member discusses that project team recognizes some of the topics discussed in the session may bring up difficult memories or experiences and informs participants that mental health and trauma resources will be shared with attendees after the session.*

What questions do you have before we start?

Is it ok if I start the recording?

Question Guide

Question	Minutes
Before we jump into our discussion let's quickly introduce ourselves. We'll go around the group and ask you to share your name (it doesn't have to be real name, just what you'd like to use for this group), [the age of your child or children who have health concerns or a rare diagnosis / your age], and [something your child likes to do for fun / you like to do for fun].	5
Project staff will have prepared a slide with graphs summarizing a few characteristics of the combined group members (age symptoms began, whether have diagnosis, belong to support group) from survey completed at enrollment. Project staff will share slide on screen.<br><br>Each of you answered a few questions when you signed up to be part of a listening session. We've summarized some of that information here for everyone that signed up to be a part of tonight's group. *Moderator briefly summarizes information on slide.*<br><br>In looking over this, what stands out to you?<br>- What stands out to you about the responses that are shared among members of the group, such as X or Y?<br>- What stands out to you about the responses that differ among members of the group, such as Z?	5
We'd like each of you to share three things you feel are challenging for [children or parents of children / children and teens] with a rare diagnosis or complex health concerns? You may type this in the chat or share verbally. *Moderator will summarize those shared verbally in the chat.*	5-10
It looks like X came up for several of you, tell me more about that. – Follow with Y and Z…<br>- Could you share an example of time you experienced this challenge?<br>- How does this impact your daily or family life?<br>- How does this impact your child's healthcare?	15
Thinking about what the group has shared so far, including topics you initially shared as challenges and ones we've discussed further, if you had to pick only one challenge that you feel is most important to try to improve, which would it be? You can pick one you mentioned or something that was shared by others, or you may wish to talk about something new you've thought of.<br>- What is it about that challenge that makes it most important to you?	15
If topics around ethics and genomics arose organically in the previous discussion you may not need to ask these specifically. If they have not already come up, please ask.<br><br>- Has genetic testing played a role in any of the challenges you've discussed above?<br>- What other challenges have you experienced around genetic testing?<br><br>- Thinking about the topics the group has been discussing, what ethical dilemmas or questions have these presented? *(may define ethical dilemma as questions of right or wrong or with conflicting viewpoints, or give examples)*<br>- What other ethical dilemmas or challenges have you experienced in your child's health journey?	10
What have you felt thankful for along your journey with these health concerns or rare disease?<br>- What about that experience has been helpful to you?	10
Tonight, we've talked about your experiences with health concerns or a rare diagnosis [for your child] and what might be shared across your experiences. Our goal has been to learn from you about what is challenging, what is helpful, and what you feel is important to try to improve through research. What have we missed in our discussion? What else would you like us to know?<br><br>*Before ending ask note-taker if anything they would like to follow-up on or ask a question about.*	10
