# Supplementary material for: Developing a community-led rare disease ELSI research agenda
Source: Orphanet J Rare Dis. 2024 Jan 22;19:23. doi: 10.1186/s13023-023-02986-x (PMC10801933; doi:10.1186/s13023-023-02986-x)
Supplement: Supplementary file 1 — Additional file 1: Example listening session recruitment material posted on social media that demonstrates a word cloud of terms related to rare disease developed by the Recruitment Team [file 13023_2023_2986_MOESM1_ESM.pdf]

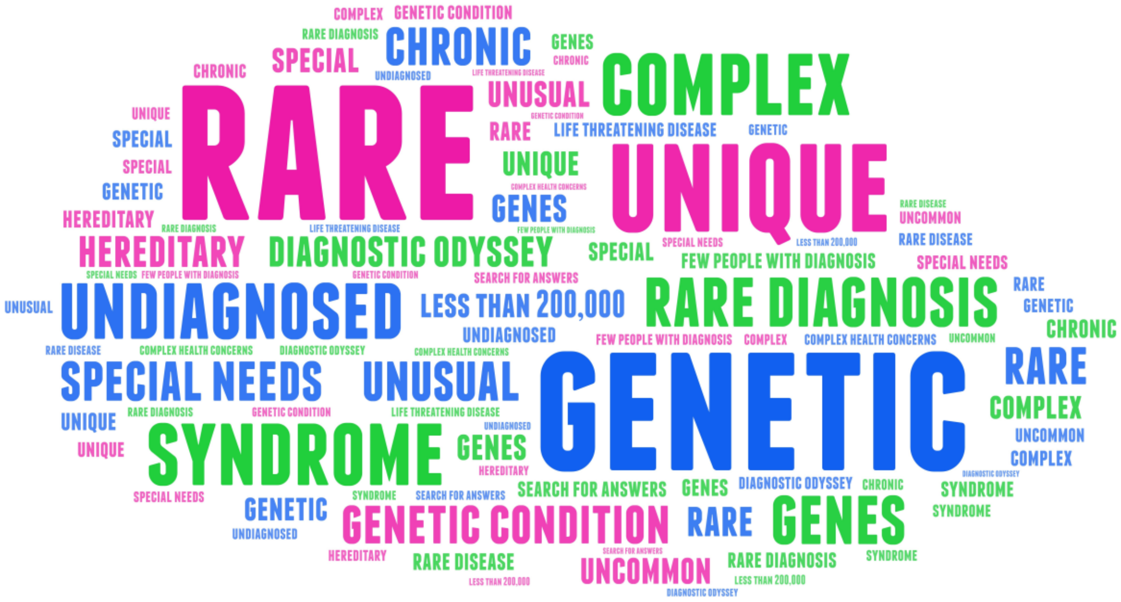

Do you care for a child with… Are you a teen with…

a rare condition?
a genetic diagnosis?
complex health concerns?
symptoms with an unknown cause?

Share your voice and shape future research to inform care for patients and families!

Children's Mercy KANSAS CITY | Research Institute

Additional file 1. Example listening session recruitment material posted on social media that demonstrates a word cloud of terms related to rare disease developed by the Recruitment Team.
